# Lipids in salicylic acid-mediated defense in plants: focusing on the roles of phosphatidic acid and phosphatidylinositol 4-phosphate

**DOI:** 10.3389/fpls.2015.00387

**Published:** 2015-05-28

**Authors:** Qiong Zhang, Shunyuan Xiao

**Affiliations:** ^1^Institute for Bioscience and Biotechnology Research, University of MarylandRockville, MD, USA; ^2^Department of Plant Sciences and Landscape Architecture, University of MarylandRockville, MD, USA

**Keywords:** plant defense signaling, lipid signaling, salicylic acid, phosphatidic acid, phosphatidylinositol 4-phosphate, phospholipase D, phospholipase C, biphasic generation of ROS

## Abstract

Plants have evolved effective defense strategies to protect themselves from various pathogens. Salicylic acid (SA) is an essential signaling molecule that mediates pathogen-triggered signals perceived by different immune receptors to induce downstream defense responses. While many proteins play essential roles in regulating SA signaling, increasing evidence also supports important roles for signaling phospholipids in this process. In this review, we collate the experimental evidence in support of the regulatory roles of two phospholipids, phosphatidic acid (PA), and phosphatidylinositol 4-phosphate (PI4P), and their metabolizing enzymes in plant defense, and examine the possible mechanistic interaction between phospholipid signaling and SA-dependent immunity with a particular focus on the immunity-stimulated biphasic PA production that is reminiscent of and perhaps mechanistically connected to the biphasic reactive oxygen species (ROS) generation and SA accumulation during defense activation.

## 1. Introduction

Plants have evolved multilayered preformed and inducible defense mechanisms to fight against various pathogens. In most cases, plant defense responses are induced upon recognition of non-adapted and adapted pathogens by a two-branched innate immune system (Jones and Dangl, 2006). For the first branch, defense is triggered upon recognition of conserved pathogen- or microbial- associated molecular patterns (PAMPs or MAMPs) by plant cell surface-localized pattern recognition receptors (PRRs) thus it is referred to as PAMP/MAMP-triggered immunity (PTI/MTI). For the second branch, plants employ cell-surface receptor-like proteins or intracellular nucleotide binding site leucine-rich-repeats (NB-LRR) proteins (genetically defined as R proteins) to recognize effectors that are secreted by pathogens to suppress PTI and promote pathogenesis, thereby inducing defense responses termed effector-triggered immunity (ETI) (Jones and Dangl, 2006). PTI and ETI are believed to be evolutionarily inter-related and mechanistically interconnected, as both involve activation of an overlapping array of downstream defense responses including *PR* gene expression, reactive oxygen species (ROS) production and callose deposition via conserved interwoven signaling pathways that are regulated by salicylic acid (SA), jasmonic acid (JA), and ethylene (C_2_H_4_) (Bari and Jones, 2009; Pieterse et al., 2012), despite clear branch-specific differences in crosstalk directionality and outcome strength.

SA, the best-studied small phenolic phytohormone, plays a major role in mediating defense against biotrophic and hemi-biothrophic pathogens that rely on living host cells for establishing infection (Vlot et al., 2009). Cellular SA accumulation constitutes an early signaling event during PTI and ETI and is essential for induction of defense responses. This step requires components including EDS1, and its homologous & interacting partners PAD4 and SAG101 (positive regulators of SA signaling) (Wagner et al., 2013), as well as SID2 (required for 90% stress-induced SA biosynthesis) (Wildermuth et al., 2001) and EDS5 (required for SA transport from the chloroplast to the cytoplasm) (Serrano et al., 2013). Elevation of SA level is perceived by SA receptors NPR3 and NPR4, which leads to degradation of NPR1, the master regulator of SA, in the infected cell, resulting in effector-triggered cell death; whereas NPR1 accumulates in neighboring cells to promote cell survival and SA-mediated resistance (Fu and Dong, 2013; Yan and Dong, 2014). In addition, SA signaling engages a feedback circuit to amplify defense responses (Wiermer et al., 2005), which is negatively regulated by EDR1, a MAPKKK (Frye et al., 2001; Xiao et al., 2005).

While protein components are essential for plant immunity and have been extensively studied, important roles for signaling lipids and their metabolizing enzymes in plant immunity have also been observed but relevant studies on the latter lag far behind. Even less is known about the possible mechanistic connection between lipid signaling and SA-dependent defense responses. In this mini-review, we will examine recent literatures on the “lipid-SA” connection with a focus on discussing how two phospholipids, i.e., phosphatidic acid (PA) and phosphatidylinositol 4-phosphate (PI4P), and the related enzymes [phospholipase D (PLD), phospholipase C (PLC), diacylglycerol kinase (DGK), and phosphatidylinositol-4-kinases (PI4Ks)] are implicated in SA signaling during PTI and ETI. For more detailed information on the biochemistry of these phospholipids and their metabolizing enzymes, and their roles in plant stress responses, we recommend several excellent reviews (Wang, 2004, 2014; Bargmann and Munnik, 2006; Arisz et al., 2009; Li et al., 2009; Munnik and Nielsen, 2011; Testerink and Munnik, 2011).

## 2. Role of PA in defense: both positive and negative

Being the simplest phospholipid class, PA has rather versatile functions: it is not only a central intermediate in glycerolipid biosynthesis but also a signaling molecule involved in regulating cellular processes such as lipid metabolism, signal transduction, cytoskeletal rearrangements, and vesicular trafficking. The concentration of PA is normally very low in plant tissues and can be induced rapidly by various stimuli. This signal-induced PA is mainly produced via two distinct enzymatic pathways. The first route is accomplished in a two-step enzymatic process that involves generation of diacylglycerol (DAG) from inositol phospholipids by PLC, followed by production of PA through phosphorylation of DAG by DGK. The other route engages PLD to produce PA through direct hydrolysis of phospholipids such as phosphatidylcholine and phosphatidylethanolamine by removing their head groups (Testerink and Munnik, 2011). In the presence of primary alcohols such as n-butanol, PLD prefers alcohols over water molecules to produce phosphatidyl alcohols instead of PA through a reaction called transphosphatidylation (Yang et al., 1967). This unique property allows researchers to easily monitor PLD activity to study the role of PLD-derived PA under different conditions, and distinguish PA produced by PLD from that produced by PLC-DGK (Arisz et al., 2009). Conceivably, PA derived from the above-mentioned two pathways may possess structural diversity (fatty acyl chain length and degree of saturation) as well as distinct spatiotemporal characteristics at the tissue, cell or subcellular level. Hence, a multifaceted role of PA in cellular signaling is anticipated and can be attributed largely to the properties of the specific enzymes that produce different pools of signaling PA with spatiotemporal specificity.

A potential role of PA in plant defense was inferred by the observations that transcription of plant *PLC*, *DGK* or *PLD* genes and/or their protein enzymatic activities were induced to higher levels upon pathogen infection or elicitor treatment in rice (*Oryza sativa*) (Young et al., 1996; Yamaguchi et al., 2003, 2005), tomato (*Solanum lycopersicum*) (van der Luit et al., 2000), tobacco (*Nicotiana tabacum*) (Suzuki et al., 2007), and Arabidopsis (*Arabidopsis thaliana*) plants (de Torres Zabela et al., 2002). Subsequent genetic or biochemical studies provided more definitive evidence to support differential or even opposing roles of PA in regulation of plant defense response under different pathocontexts (Supplemental Table 1). In tomato suspension-cultured cells expressing the *Cf-4* resistance gene, treatment of the cognate pathogen effector Avr4 rapidly induced accumulation of PA, via the PLC-DGK route (De Jong et al., 2004). Further studies showed that silencing of the tomato *SlPLC4* gene impaired Cf-4/Avr4-induced HR and resulted in increased susceptibility of *Cf-4* plants to *Cladosporium fulvum* expressing *Avr4* (Vossen et al., 2010). Interestingly, silencing of *SlPLC6* in tomato did not affect Cf-4/Avr4-induced HR, but compromised resistance mediated by *R* genes like *Cf-4*, *Ve1* or *Pto/Prf*. These observations demonstrate that PLC-DGK-derived PA probably acts as a positive regulator of ETI. In Arabidopsis, two recent studies have established a positive role for PLD-derived PA in basal defense and non-host resistance. These studies showed that abrogation of PLD-derived PA by n-butanol in Arabidopsis compromised both basal (cell-wall-based) resistance to non-adapted powdery mildew pathogens and RPM1(an NB-LRR)/AvrRpm1(the cognate effector)-triggered immunity (Pinosa et al., 2013; Johansson et al., 2014). Genetic analysis of Arabidopsis mutants identified *At*PLDδ, one of the 12 *At*PLDs, to be the only isoform that contributes to penetration resistance against non-adapted powdery mildew (Pinosa et al., 2013), yet no single PLD isoform was found to be responsible for RPM1/AvrRpm1-triggered immunity, highlighting functional redundancy among different *At*PLD isoforms (Johansson et al., 2014).

Interestingly, while so far there is no evidence for PLC-DGK-derived PA in negative regulation of plant defense, genetic depletion of specific PLD isoforms in tomato, rice, and Arabidopsis resulted in elevated defense responses. These genes include *SlPLDβ1* (its silencing resulted in priming for a subset of defense responses in tomato cells treated with elicitors) (Bargmann et al., 2006), *OsPLDβ1* (its silencing in rice resulted in enhanced resistance to multiple pathogens) (Yamaguchi et al., 2009), and *AtPLDβ1* (its expression was suppressed by SA and genetic depletion led to elevated levels of SA, ROS, and enhanced resistance to virulent *P. syringae*) (Zhao et al., 2013).

Apparently, further studies are required to gain more mechanistic insight into how PA derived from different PLDs might oppositely regulate defense responses in plants. In the following section, we carefully examined the temporal kinetics of PA generation and manifestation of defense response in searching for possible intrinsic causal relationships between PA and SA signaling.

## 3. A biphasic connection between PA and SA signaling

Several earlier studies showed that both pathogen- or elicitor-induced production of ROS, SA, and C_2_H_4_ exhibited a biphasic pattern (Alvarez et al., 1998; Mur et al., 2000, 2003, 2008, 2009). Interestingly, as seen from the data summarized in Supplemental Table 1 a biphasic PA production upon PAMP/effector treatment has also been either inferred from increased PLC and/or PLD gene/enzyme activities or direct detection. Since (i) PA production appeared to occur earlier than ROS generation (Sang et al., 2001; De Jong et al., 2004; Park et al., 2004), (ii) PA was indeed shown to induce ROS production by activating the NADPH oxidase RbohD (Zhang et al., 2009; Tetiana et al., 2013) which is the main NADPH oxidase responsible for H_2_O_2_ generation during PTI (Kadota et al., 2014; Li et al., 2014), and (iii) ROS generation could lead to SA level elevation (Lamb and Dixon, 1997; Chamnongpol et al., 1998; Mur et al., 2009), we propose that PA likely functions as an important initial signal in the biphasic defense signaling waves.

### 3.1. A potential biphasic PA production during PTI?

In tomato suspension-cultured cells, formation of PA (by *Sl*PLC) was detected within a few minutes after application of elicitors N,N,N,N-tetraacetylchitotetraose, xylanase, and flg22, which coincided with H_2_O_2_ production (van der Luit et al., 2000; Bargmann et al., 2006). Whether treatment of these PAMPs triggered the second wave of PA and ROS generation was not known in these circumstances since the measurement was restricted to the first 2 h post-elicitation which may preclude the second wave of PA and ROS production at later time points. Notably, using rice suspension-cultured cells, Yamaguchi and colleagues did detect a biphasic induction of ROS that coincided with (and probably was preceded by) *Os*PLC and/or *Os*PLD activation, in which case the first peak at 20 min was associated with the activation of both *Os*PLC and *Os*PLD whereas the second peak at 120 min was associated mostly with the activation of *Os*PLD, after application of a PAMP-like elicitor N-acetylchitooligosaccharide (Yamaguchi et al., 2005). Exogenous application of PA could induce ROS generation by itself, suggesting that ROS production was induced by enzymatic activities of *Os*PLC and/or *Os*PLD. Thus, although no direct quantification of PA was conducted in this study, a biphasic PA production (as a result of *Os*PLD activation) before the biphasic ROS production was anticipated. It seems clear that PAMPs could trigger the first phase of PA production, but whether or not they can also induce the second phase remains to be determined.

### 3.2. A biphasic PA production during ETI

Using transgenic tobacco cells expressing the tomato *Cf-4*-resistance gene as a model system, it was found that within 2 min after challenge with the fungal effector Avr4, a largely *Sl*PLC-DGK-dependent PA production was detected, followed by an oxidative burst a few minutes later (De Jong et al., 2004). Since no measurement for PA or ROS was done beyond 30 min, occurrence of the second wave of PA and ROS production, though anticipated, was not determined. However, because silencing of *SlPLC4* impaired Cf-4-dependent resistance and silencing of *SlPLC6* compromised several R-mediated resistance (Vossen et al., 2010), one can infer that the initial PA production is essential for ROS generation, HR and defense during ETI. It is worth pointing out that Cf-4 and Avr4, which are genetically defined as R and Avr, respectively, may arguably qualify for a PRR and a PAMP, respectively (Thomma et al., 2011). If so, Cf-4/Avr4 interaction-induced PA production before ROS generation would also render support to a biphasic PA production as an early signaling step of PTI.

Supporting this notion, Andersson and colleagues found that the first detectable wave of PA accumulation (via the PLC-DGK route) started in ~60 min which was followed by a second wave of PA production (via PLD route) occurred around 3~4 h after application of dexamethasone to induce expression of *AvrRpm1* or *AvrRpt2* as transgenes in Arabidopsis plants containing the cognate receptor (Andersson et al., 2006). Given that PTI and ETI signaling mechanisms are believed to be interconnected, it should not be a surprise that an early signaling step conserved for PTI and ETI is channeled through PA production. Recent findings that PAD4 functions upstream of SA in defense signaling during both PTI and ETI (Tsuda et al., 2009; Kim et al., 2014), and that SA can further up-regulate expression and signaling of PRRs (Zhang et al., 2014) provide likely mechanistic connection between PTI and ETI concerning PA production.

### 3.3. Possible biphasic PA-ROS-SA signaling amplification?

While a robust biphasic production of ROS or SA was described in Arabidopsis (Shapiro and Gutsche, 2003), potato (Yoshioka et al., 2001), and tobacco plants (Lamb and Dixon, 1997; Mur et al., 2000) during PTI/ETI, a clear biphasic PA production in a similar time window from elicitation to manifestation of immune response was also observed in Arabidopsis (Andersson et al., 2006), and rice cells (Yamaguchi et al., 2005). Unfortunately, there were no time-course studies in which levels of PA, ROS, and SA were measured using the same pathosystem, making it impossible to directly assess the timing of these signaling events because of the differences in the experimental systems reported (plant species, cell types, and pathogens/elicitors). However, given that ROS production and SA accumulation are tightly linked and form a self-amplifying feedback circuit during defense signaling (Mur et al., 2009; Vlot et al., 2009), we can envision that PAMP/effector-triggered PA production constitutes an important early signaling step that results in the first wave of ROS production, which in turn triggers SA biosynthesis, forming the first signaling phase that potentiates the second phase. Such signaling waves may also involve other signaling molecules such as calcium fluxes (Grant et al., 2000) and C_2_H_4_ (Mur et al., 2008).

**Figure 1:**
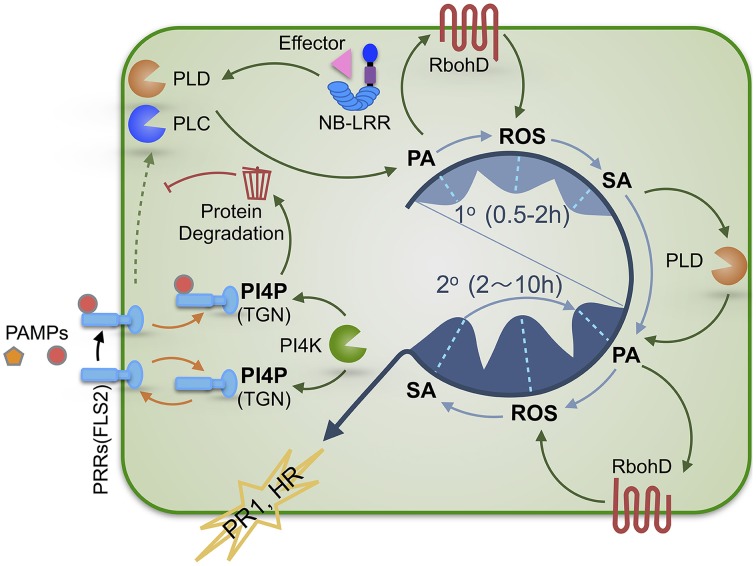
**A schematic illustration of the regulatory roles of PA and PI4P in SA-dependent plant defense signaling**. Plants activate immune responses upon recognition of PAMPs or effectors by PRRs or NB-LRR immune receptors, respectively. Bioactive PA and PI4P play distinct roles in regulating defense signaling. PA production via immunity-activated PLC and/or PLD is required for SA-dependent defense activation and exhibits a biphasic pattern (1° & 2°) that precedes the kinetically similar biphasic ROS generation and SA accumulation. Therefore, we propose that these three signaling molecules are sequentially interconnected with PA most likely being the initial signal of the PA-ROS-SA signaling module. The first wave (1°) of PA-ROS-SA signal amplification (occurring during PTI and ETI) may potentiate the second wave (2°) of PA-ROS-SA signal amplification (occurring mostly during ETI or strengthened PTI), constituting a tunable signaling module for defense in plant cells. PI4P derived from PI4KIIIβ1 and PI4KIIIβ2 functions to maintain the homeostasis of PRRs via facilitating its recycling and/or degradation, thereby preventing inappropriate activation of PTI in the absence of pathogens and allowing measured PTI signaling upon pathogen attack. PAMPs, pathogen-associated molecular patterns; PTI, PAMP-triggered immunity; ETI, effector-triggered immunity; PRR, pattern recognition receptor; NB-LRR, nucleotide binding site leucine-rich-repeats; PA, phosphatidic acid; PI4P, phosphatidylinositol 4-phosphate; TGN, trans-Golgi network; PLC, phospholipase C; PLD, phospholipase D.

Based on the evidence from multiple studies described above and summarized in Supplemental Table 1 we propose a model to illustrate biphasic PA-ROS-SA signaling during plant defense activation (Figure 1). The main points of the model are as follows: (i) The first wave is rapid and transient, and is attributable to PTI and/or ETI; whereas the second wave occurs in plant cells undergoing ETI or ectopically strengthened PTI (i.e., suspension-cultured cells treated with high-concentration of PAMPs). (ii) The signaling order is probably from PA (mainly from the PLC-DGK route) to ROS (De Jong et al., 2004; Park et al., 2004), and from ROS to SA (Lamb and Dixon, 1997; Chamnongpol et al., 1998; Mur et al., 2009) in the first wave based on time sequence and some known mechanistic connections. (iii) Elevated SA in the first wave above a threshold level plays an essential role in potentiation (priming) of the second wave of PA (mainly produced by PLDs), ROS and SA production through multi-layered positive feedback amplification circuits where EDS1/PAD4/SAG101 may be essential components required for SA signaling and PTI-ETI connection (Kim et al., 2014; Zhang et al., 2014). Conceivably, the spatiotemporal kinetics and amplitude of the biphasic defense signal amplification may vary under different pathocontexts, which may at least partially account for the discrepancies in the results from different studies (Supplemental Table 1). Nevertheless, the biphasic PA-ROS-SA signal amplification, together with production of other signaling molecules such as nitric oxide and C_2_H_4_ (Mur et al., 2008, 2009), likely orchestrates the eventual development of HR and other defense responses in many cases.

## 4. PI4P chimes in to put a brake on and fine-tune PTI

One critical question one may ask is why plant defense signaling is biphasic but not monophasic or incremental. A logical explanation is that there must be concomitant or instantaneous negative regulation on it. Indeed, PAMP-elicited or EDS1-dependent defense signaling has been demonstrated to be tightly regulated by a number of negative regulators. These include the E3-ubiquitin ligase PUB13 (Lu et al., 2011; Zhou et al., 2014), the Ca^2+^/calmodulin-binding transcription factor SR1 (Du et al., 2009) and a MAPKKK EDR1 (Frye et al., 2001; Xiao et al., 2005). Interestingly, recent studies showed that PI4KIIIβ1, PI4KIIIβ2, and their product PI4P negatively regulated SA signaling via modulating homeostasis of FLS2, a PRR that recognizes flagellin (a PAMP from bacteria) (Antignani et al., 2015), providing a possible braking mechanism for PTI.

PI4Ks catalyze the phosphorylation of phosphatidylinositol at the 4th -OH position of its inositol head group to produce PI4P, the precursor of PI(4,5)P_2_. PI4Ks are divided into two major types, II and III, according to their sizes and sensitivities to pharmacological treatments. Based on sequence and structure similarities, type III PI4Ks are further grouped into two subfamilies, α and β (Mueller-Roeber and Pical, 2002).

In an earlier report, PI4KIIIβ1 and PI4KIIIβ2 were shown to be negative regulators of SA signaling in Arabidopsis, as the *pi4kIIIβ1β2* double mutant plants accumulated high levels of SA and ROS, constitutively expressed the *PR-1* gene and showed enhanced resistance to *P. syringae* (Šašek et al., 2014; Antignani et al., 2015). Interestingly, PI4KIIIβ1 and PI4KIIIβ2 were reported to interact with a small GTPase RabA4B in the Arabidopsis root tip to regulate polarized expansion of root hair cells (Preuss et al., 2006). Recently, Antignani and colleagues showed that both RabA4B and PI4P interacted with PUB13 and the authors proposed that PI4KIIIβ1 and PI4KIIIβ2 were recruited by RabA4b to assist in the enrichment of PI4P at the trans-Golgi network (TGN) for (i) proper sorting of FLS2 via recycling it back to the plasma membrane, or (ii) promoting FLS2 turnover by recruiting PUB13 to FLS2 (Antignani et al., 2015). Thus, PI4KIIIβ1 and PI4KIIIβ2, and more relevantly PI4P, function to negatively regulate SA signaling by maintaining FLS2 homeostasis. Meanwhile, PI4P can be converted to PI(4,5)P_2_ which can activate PLDβ (Zheng et al., 2002), a genetically defined negative regulator of SA signaling in Arabidopsis (Zhao et al., 2013). Thus, PI4P may also exert its negative role via stimulating PLDβ indirectly. Intriguingly, another study showed that PI4Ks could be activated within 2 min upon SA treatment in Arabidopsis suspension-cultured cells, preceding the activation of PLD (45 min after SA treatment) (Krinke et al., 2007). Whether PI4KIIIβ1 and PI4KIIIβ2 were among the activated PI4Ks is not known. Regardless, PAMP-elicitation may lead to recruitment of PI4Ks to the TGN and subsequent local enrichment of PI4P, which may assist in recruiting PUB13 and facilitating its role in targeted degradation of PRRs, thereby down-regulating PTI signaling, resulting in a measured initial wave of PA-ROS-SA production for potentiating the second defense signaling wave (Figure 1).

## 5. Conclusions and perspectives

Increasing evidence from biochemical and genetic studies suggests that PA from different sources may play distinct roles in plant immune responses, while PI4P may negatively regulate PTI signaling. Interestingly, as demonstrated by or inferred from multiple studies, immunity-stimulated PA production exhibits a biphasic pattern that is reminiscent of the biphasic ROS generation and SA accumulation. Hence, it appears likely that a major role of PA in plant immunity is to initiate and orchestrate biphasic amplification of ROS- and SA-dependent signaling leading to downstream defense responses. However, because of the intrinsic complexity of such regulatory mechanisms, the diverse experimental systems used, the genetic redundancies, and the difficulty in measuring the (sub)cellular levels of target lipid molecules, results from many individual studies are either descriptive in nature or fragmental. Future studies will be directed to defining the roles of signaling phospholipids and their metabolizing phospholipases in plant immunity by (i) using higher-order genetic mutants to circumvent functional redundancy, (ii) using novel tools and technologies to investigate the spatiotemporal dynamics of target molecules at the subcellular level, and (iii) studying of multiple defense signaling molecules in the same pathosystem.

### Conflict of interest statement

The authors declare that the research was conducted in the absence of any commercial or financial relationships that could be construed as a potential conflict of interest.
